# Role of sphingosine 1-phosphate (S1P) in sepsis-associated intestinal injury

**DOI:** 10.3389/fmed.2023.1265398

**Published:** 2023-09-08

**Authors:** Gehui Sun, Bin Wang, Hongquan Zhu, Junming Ye, Xiaofeng Liu

**Affiliations:** ^1^Gannan Medical University, Ganzhou, Jiangxi, China; ^2^The First Affiliated Hospital of Gannan Medical University, Ganzhou, Jiangxi, China; ^3^Department of Critical Care Medicine, The First Affiliated Hospital of Gannan Medical University, Ganzhou, Jiangxi, China; ^4^Suzhou Medical College of Soochow University, Suzhou, Jiangsu, China; ^5^Department of Emergency, The First Affiliated Hospital of Gannan Medical University, Ganzhou, Jiangxi, China

**Keywords:** S1P, sepsis, S1PRs, signaling, intestinal epithelial barrier, immune

## Abstract

Sphingosine-1-phosphate (S1P) is a widespread lipid signaling molecule that binds to five sphingosine-1-phosphate receptors (S1PRs) to regulate downstream signaling pathways. Sepsis can cause intestinal injury and intestinal injury can aggravate sepsis. Thus, intestinal injury and sepsis are mutually interdependent. S1P is more abundant in intestinal tissues as compared to other tissues, exerts anti-inflammatory effects, promotes immune cell trafficking, and protects the intestinal barrier. Despite the clinical importance of S1P in inflammation, with a very well-defined mechanism in inflammatory bowel disease, their role in sepsis-induced intestinal injury has been relatively unexplored. In addition to regulating lymphocyte exit, the S1P-S1PR pathway has been implicated in the gut microbiota, intestinal epithelial cells (IECs), and immune cells in the lamina propria. This review mainly elaborates on the physiological role of S1P in sepsis, focusing on intestinal injury. We introduce the generation and metabolism of S1P, emphasize the maintenance of intestinal barrier homeostasis in sepsis, and the protective effect of S1P in the intestine. We also review the link between sepsis-induced intestinal injury and S1P-S1PRs signaling, as well as the underlying mechanisms of action. Finally, we discuss how S1PRs affect intestinal function and become targets for future drug development to improve the translational capacity of preclinical studies to the clinic.

## 1. Introduction

Sepsis is a life-threatening organ dysfunction syndrome caused by a dysregulated host response to infection, Sepsis is clinically characterized by severe infection, systemic inflammatory response, and organ dysfunction, and is associated with high morbidity and mortality (1, 2). In ICU ward, clinicians usually use SOFA score to judge the prognosis of patients, the international Sepsis3.0 definition proposes that sepsis can be diagnosed when the patient reaches “infection and SOFA score ≥ 2” (3). An estimated 31.5 million cases of sepsis and 19.4 million cases of severe sepsis have been reported worldwide. Sepsis accounts for potentially 5.3 million deaths annually and the mortality rate among patients with sepsis in ICU Ward can reach 41.9% (2, 4). Incidence of sepsis will continue to rise with the increasing ages of the populations and corresponding rise in the underlying diseases (5). Neglect in the disparities in sepsis cases in developing and middle-income countries and the lack of epidemiological data on sepsis in low- and middle-income countries can lead to underestimation of incidence of sepsis and associated mortality (2, 4). The intestine is the largest immune barrier organ in the human body and is the home for a microbiome composed of more than 100 trillion species of bacteria. The host and the bacteria are mutually beneficial and symbiotic, under normal physiological conditions, the intestinal environment maintains its balance. The intestinal barrier can protect the organism from intestinal microbes and toxins (6, 7). The earlier studies have indicated that the gut is the driving force for multiple organ dysfunction in critical illness (8). Intensive research on sepsis has demonstrated intestinal barrier dysfunction to be a common complication in sepsis (9). Intestinal injury is an early marker in the development of systemic inflammatory response syndrome and multiple organ failure (MOF) (10). Sepsis causes disturbance of the gastrointestinal microenvironment, causing an imbalance between the host and the gut microbiota, which further leads to compromised intestinal barrier function and reduced intestinal immunity (11, 12). Meanwhile, studies have shown that sepsis is associated with a higher mortality rate caused by gastrointestinal injury (13).

Sphingosine-1-phosphate (S1P) is a lipid substance that is widely present in human body fluids, tissues, and cells and is responsible for conveying intercellular information as a first messenger (14). During the pathogenic process of sepsis, S1P maintains the integrity of vascular endothelial cells (ECs), promotes lymphocyte circulation, reduces systemic inflammatory responses, as well as protects the integrity of the intestinal barrier (15, 16). With the development of drugs targeting S1P and agonists and antagonists of Sphingosine-1-phosphate receptors (S1PRs), several therapeutic strategies such as FTY720, an agonist of S1PRs, which improves systemic conditions and reduces local inflammation in the intestine of mice have been proposed for treating inflammatory bowel disease (17, 18). Currently, available clinical trials have demonstrated decreased plasma concentrations of S1P in patients with sepsis compared with normal individuals (19), and have also indicated that the S1P levels are further lowered with increasing severity of sepsis (20, 21). Recent studies have confirmed that S1P alleviates LPS-induced intestinal epithelial cell injury, maintains the colonic mucosal barrier, and prevents intestinal injury during sepsis (22). S1P binding to different S1PRs activates downstream signaling pathways involved in cell proliferation, migration, apoptosis, immune regulation, anti-inflammation, and information transmission processes, showing multiple effects (14, 23). Studies have shown that the intestine is an immune organ and S1P can attenuate sepsis intestinal injury by regulating immune responses (24). Therefore, understanding the role of S1P in the septic gut is of great significance for the treatment of sepsis and sepsis-associated intestinal injury (23, 25). In this review, we have summarized the recent research progress on protective effects of the epithelial barrier and potential therapeutic targets of S1P in sepsis-associated intestinal injury.

## 2. S1P generation and metabolism

**FIGURE 1:**
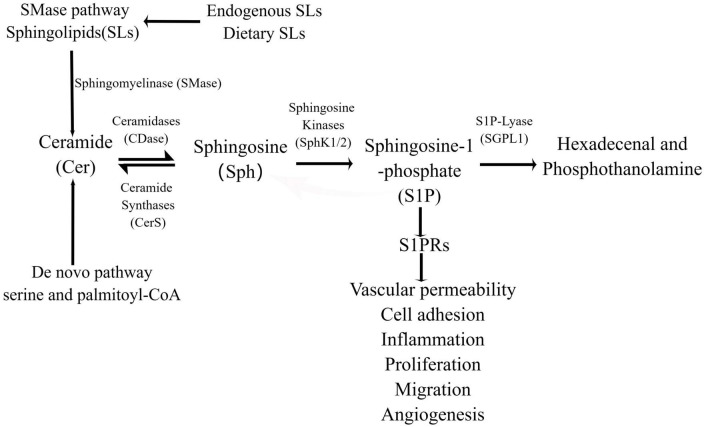
The production and function of S1P in the intestine. S1P is generated in the intestine through SMase pathway, *de novo* synthesis and rescue pathway, and the *de novo* synthesis pathway is the main one. In the SMase pathway, bioactive SLs are hydrolyzed to ceramides by SMases, and in the endoplasmic reticulum, serine and palmitoyl-coA are synthesized by the *de novo* pathway. Ceramide is transported to the Golgi apparatus to generate sphingosine under the action of ceramidase, S1P under the catalysis of intracellular SphK1/2, and hexadecenal and phosphoethanolamine under the action of SPL. In addition, sphingosine can also be acylated by ceramide synthetase to form ceramides in the ER, a process known as the “salvage pathway.”

Sphingosine 1-phosphate is synthesized either by a *de novo* pathway from serine and palmitoyl-CoA or produced by a the sphingomyelinase (SMase) pathway from the ubiquitous membrane lipid sphingomyelin (SM), ceramide (Cer), and sphingosine (Sph) are intermediates the sphingomyelinase in both pathways (26). Sph can be recovered by acylation, which is called “salvage pathway,” leading to regeneration of Cer (27, 28). This is followed by hydrolysis of Cer to generate Sph, and finally Sph generates S1P following the action of sphingosine kinases (SphKs) in a process that occurs in the cell membrane and cytoplasm (29) (Figure 1, By Figdraw). Ultimately, S1P is either cleaved by S1P lyase or gets dephosphorylated to Sph by S1P phosphatase (30, 31). SMase pathway begins with sphingolipids (SLs), which are ubiquitous structural components in cell membranes, SLs metabolites affect cell apoptosis, cell growth and cell migration (32). Endogenous SLs play crucial roles at multiples stages in cell biological processes and human health (33). With the development of research, it has been found that bioactive SLs, e.g., SM, Sph, Cer, S1P, and ceramide-1-phosphate may derive from dietary SLs ingested through the diet (34). Dietary SLs are ingested and absorbed in the gastrointestinal tract, affecting immune activation status, contributing to pro-inflammatory and anti-inflammatory immune responses, and can be used by cells to regulate growth, differentiation, apoptosis, and other functions (15, 35). Some studies have shown that exogenous SLs may dampen both acute and chronic inflammatory responses in cell and animal models (36, 37). According to earlier studies conducted in the last century, consumption of SLs in the United States has been estimated to be 0.3–0.4 g/day (35). SM in milk has been shown to promote gut developmental maturation (36). Dietary SM is digested by intestinal alkaline SMase (alk-SMase) and neutral ceramidase (n-CDase), and finally hydrolyzed into Cer, choline phosphate, Sph and fatty acids in the small intestine (34). Sph can be completely absorbed into intestinal mucosal cells and transformed into S1P, which is transported to lymph circulation and blood circulation together with chyle particles (38). The metabolism of SLs is affected by the type of fatty acid in diet, vitamin B6, vitamin C, vitamin D, and vitamin K (34, 39).

Sphingosine 1-phosphate is mainly derived from red blood cells, lymphatic endothelial cells and platelets (40), and S1P concentration in blood and lymph fluid is much higher than that in lymphatic tissue, forming S1P gradient (29). Sph is converted to S1P in the intestinal mucosa, where SPHK1/2 and SPL are highly expressed, and where SPL content is also higher than in any other tissue (41, 42). S1P is expressed in epithelial cells of the small intestine and colon mucosa, but the level in the small intestine is more than 2-fold higher than that in the colonic mucosa (43). Due to the abundant blood flow, lymph and lymphocytes in the intestine, which can digest food, and the presence of a large number of intestinal microorganisms, studies have found that dietary SM is mainly destroyed into sphingosine and sphinganine in the jejunum through a large amount of alk-SMase and nCDase released by the intestinal epithelium and liver, which are rapidly absorbed and metabolized by intestinal mucosal cells to generate S1P (44). SL metabolites (including S1P) can also be produced by gut microbes and directly affect host metabolic pathways, for example, *Bacteroides fragilis* (15, 45).

Sphingosine 1-phosphate is a bioactive sphingolipid that is present in high-density lipoprotein (HDL), and the main binding carrier is apolipoprotein M (apoM). In plasma, 60% of S1P is normally bound to apoM and 40% binds to albumin (46). S1P needs to bind to these carriers to be transported in the biological fluids (47). Moreover, S1P, being a small polar phospholipid, is transported through the lipid bilayer in a paracrine or autocrine manner, with the aid of the transporter ATP-binding cassette (ABC) transporters and spinster homolog 2 (SPNS2) (48).

Sphingolipids are metabolized in the intestine and are primarily found in the mucosal cells of the small intestine and colon, where their metabolites are absorbed and enter the blood circulation or lymph (39). S1P, which is widely found in the blood and lymph, is more abundant in the gut than in other tissues, and influences the immune status of the intestines (29, 43). The maximum concentration of S1P in circulation cells is found in erythrocytes, EC, immune cells, and platelets, as the activities of S1P lyase and phosphatase are weak within erythrocytes, which ensure that the high concentrations of S1P are maintained (49). Lymphatic ECs are the main source of S1P in the lymph (29). Multiple stimuli during sepsis regulate key enzymes, thereby affecting sphingolipid biosynthesis and metabolism, resulting in restricted S1P production (29). The finding in an earlier study, according to which the S1P levels in the blood of patients with sepsis were lower than normal, could be attributed to the fact that apoM levels are reduced in patients with sepsis and systemic inflammatory response syndrome; because of which S1P plasma levels are also lower (50). In an experiment on sepsis in humans and baboons, it was observed that S1P levels were lower in most septic groups, when compared to normal controls, less by almost 46% in severe septic shock, and a homogeneous distribution of S1P was observed between apoM and albumin. A significant correlation was observed between S1P decline and apoM during sepsis (20). Because hepatocytes express and secrete the majority of apoM and albumin, serum S1P levels were found to decrease sharply with advanced liver disease and predict early death in patients with chronic liver disease, and low S1P levels may significantly affect the progression of multiple organ dysfunction syndrome (MODS) (51). Makoto Kurano’s experiment demonstrated that while knockout or knockdown of apoM aggravated lethality and organ damage in lipopolysaccharide (LPS) mice, apoM/S1P improved survival and protected organs by preventing apoptosis (52). While erythrocytes are the major source of S1P in plasma, during sepsis, they also contribute to iatrogenic blood loss, reduced plasma iron, suppressed erythropoietin production and shortened RBC lifespan as well as malnutrition (53), which in turn could account for the reduced S1P levels. Another important cause for reduced S1P levels in sepsis patients could be the fact that sepsis causes acute damage and dysfunction of the gastrointestinal tract, which results in insufficiency of nutritional sources for the patients (54), thereby affecting their nutritional status. However, the baboon model demonstrated no correlation between the number of erythrocytes and S1P. In contrast, platelet levels of S1P in humans closely correlate with those in the baboon sepsis (20).

Human megakaryocytes (MKs) release trillions of platelets into the circulation every day to maintain platelet levels. Studies have revealed that S1PR1 signaling continuously inhibits MKs growth, and when S1P metabolism is disrupted in the hematopoietic environment, S1PR2 signaling can further inhibit thrombosis. S1P generation is related to SphK1 activation (55).

Studies have shown that SphK1 is 15 times higher than SphK2 in normal human body, while the expression of SphK1 in platelets of sepsis patients is significantly reduced, while SphK2 has no significant change (56). The decreased activity of Sphk1 in cells will promote the production of platelets, and the metabolic pathway of S1P production is related to the disease of thrombocytopenia (55). In addition, recent studies have shown that platelets store S1P in the cytoplasm in the resting state, and after platelets are activated, this S1P pool is transported to the plasma membrane to regulate local cellular responses, in which the loss of the major facilitator superfamily transporter 2b (Mfsd2b) of S1P reduces platelet thrombosis (57, 58). In platelets, Sphk1 is responsible for S1P production, and Mfsd2b is responsible for S1P export. It has been shown that platelets release S1P to protect the heart during acute myocardial infarction (AMI), and antiplatelet strategy to preserve platelet S1P release during AMI is the most ideal strategy (59). After acute infection, the activation of coagulation and inflammation is the key cascade, leading to thromboinflammation and microthrombosis. The intestinal tract of septic mice shows strong inflammatory and thrombotic reactions (60), and severe imbalance of coagulation and anticoagulation can lead to disseminated intravascular coagulation (DIC) and death. DIC, as an extreme state of systemic coagulation activation, leads to excessive consumption of platelets due to continuous increase in coagulation activity after its appearance, and eventually forms thrombocytopenia (61). Thrombin has been considered as a potential therapeutic target for sepsis, and studying the thrombotic effects of Mfsd2b and S1P on platelets may be helpful for the treatment of the pathological process of sepsis in the future (62, 63).

It has been reported that SphK1 is 15-fold higher than SphK2 in normal humans, whereas in platelets from sepsis patients, SphK1 expression is significantly reduced with no change in SphK2 Recent studies indicate that platelets produce a large amount of S1P through *de novo* synthesis, which is released after platelet activation, thereby modulating local cellular responses (57). Thus, platelets provide S1P for a short time during sepsis, which may be associated with the activation of SphK1.

## 3. The five receptors for S1P

Sphingosine 1-phosphate, a metabolite of cell membrane sphingolipids, was reported to be a second messenger that mediates increases in intracellular calcium levels, which transmit signals by binding to S1PRs on the cell membrane surface. However, S1P was also unexpectedly found to function as an intercellular first messenger (64, 65). The five receptors for S1P are G protein-coupled receptor subtypes, and all immune cells express the unique characteristics of S1PRs, which are involved in adaptive immune cell trafficking, angiodevelopment, and homeostasis (14). For T-cells, S1PR1 signals tonically reduce T-cell chemotactic sensitivity to chemokines and thereby limit homing of blood and spleen T-cells to secondary lymphoid tissues (66).

Sphingosine-1-phosphate receptors are differentially expressed in various tissues. S1PR1 is located in most organs, and is expressed at high levels during embryonic development, vascular production and maturation, bone remodeling, and in the immune system (67). S1PR2 plays important roles in inhibiting apoptosis and proliferation, actin remodeling, and development of the cardiovascular, visual and auditory systems (23). Studies have shown that S1PR2 is highly expressed in intestinal epithelial cells (IECs) and promotes their proliferation (68). S1PR3 is found in most organ tissues, and current research focuses on cardiovascular issues, sepsis, cardiac conduction, stroke, and cancer metabolism (69). S1PR4 is mainly expressed in immune organs, and is involved in megakaryocyte differentiation and platelet development (70), and S1PR5 is expressed at high levels in spleen, skin, lung, and brain, plays a role in myelination, and is involved in nervous system expression (71, 72). The expression of S1PRs in intestinal endothelial cells is low, but a variety of S1PR-targeted drugs have high affinity for S1PR1 and S1PR5. Ozanimod, for example, has been approved for the first time in the United States, European Union and other countries for the treatment of moderate to severe active ulcerative colitis in adults, the main mechanism is to bind to S1PR1 and S1PR5 receptors. Internalization of S1PR1 reduces the ability of lymphocytes to secrete from lymphoid tissues (73). In addition, gut microbes affect gut and brain pathophysiology through the microbiome-gut-brain axis (MGBA). The gut microbiome of patients with sepsis may contribute to the pathogenesis of sepsis-associated encephalopathy (SAE). The study of septic intestinal injury will also help researchers decipher SAE (74).

**FIGURE 2:**
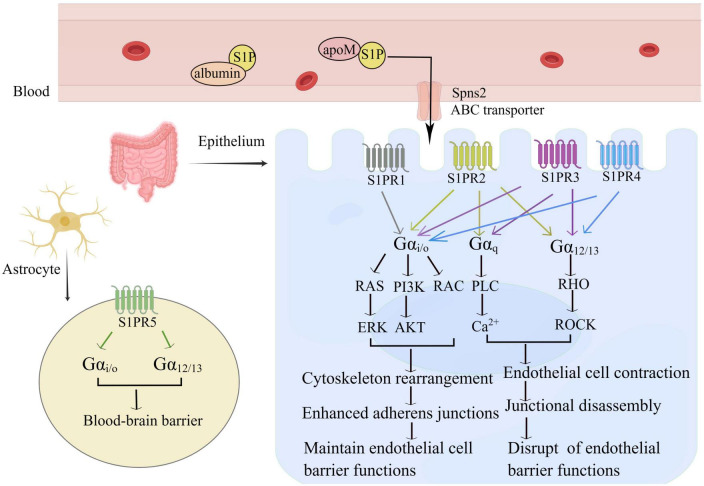
S1P maintains the intestinal intraepithelial EC barrier. S1P binds to five receptors and transmits different intracellular signals depending on the G protein-coupled G subunit and the expression pattern of each receptor in the cell type. S1PR1 is mainly coupled to G αi/o and promotes vascular stability via cytoskeleton rearrangement adherens junctions, and limits plasma and leukocyte extravasation; S1PR2 and S1PR3 are involved in G αi/o, G αq and G α12/13 pathways, S1PR4 connects G αi/o and G α12/13 pathways, interacts with Rho, leading to a diverse network of signals. S1PR2-4 can cause the contraction of vascular ECs, increase vascular permeability, and weaken endothelial barrier function. S1PR5 is mainly expressed in astrocytes and enhances the blood-brain barrier.

The S1P-S1PR signaling system contributes to the regulation of complex inflammatory processes by influencing lymphocyte trafficking and maintenance of vascular integrity. thereby S1PR1-3 is mainly expressed by ECs, and S1PR5 is expressed only by blood-brain-barrier cells (16). Under inflammatory conditions, S1PR1-3 are widely distributed, with the highest expression levels in the cardiovascular and immune systems while S1PR4 and S1PR5 are relatively less expressed (75). The downstream signaling of these receptors is very complex because they are expressed differently in ECs and are coupled to multiple G proteins. For example, S1P in the blood circulation binds to G protein-coupled receptors on the cell membrane of intestinal ECs (Figure 2, By Figdraw). After coupling with the G αi/o, S1P-S1PR1 activates the downstream Phosphoinositide 3-kinase (PI3K), phospholipase C (PLC), and Rac families, leading to Rac-dependent cytoskeletal rearrangements and cell matrix contact. It also affects the adherens junction assembly, and barrier integrity, thereby enhancing the EC barrier (71, 76). However, S1PR2 and S1PR3 bind to G αi/o, G αq, and G α_12/13_, which combined with G αq activate PLC and increase the intracellular calcium concentration, leading to vasoconstriction, decreased vascular permeability, and weakened barrier function. Both S1PR2 and S1PR3, upon binding with G α_12/13_, activate the Rho family to promote the transition of ECs to a contractile phenotype, and attenuate inter-endothelial junctions. Both pathways disrupt the capillary endothelial barrier by increasing vascular permeability. Interestingly, the two pathways mentioned above have the exactly opposite effect of combining G αi/o production (71, 77). S1PR4 and S1PR5 bind to the G α_*i/o*_, G α_12/13_ protein pathway, causing weakening of the EC barrier. The differential but overlapping expression patterns of the five receptors form the molecular basis for the different S1P functions.

## 4. The protective effect of S1P in sepsis

### 4.1. S1P protects the EC barrier in sepsis

The vascular surface is covered by a layer of ECs, which are responsible for the flow of water and solutes between the blood and the interstitium. The cells attached to the basement membrane and connected to the extracellular matrix (ECM) by focal adhesion (FA) (78). Adjacent ECs are connected by the three types of interendothelial junctions, namely, adherens junctions (AJ), tight junctions (TJ), and gap junctions (GJ) (79). Alterations in cell-cell junction expression leads to changes in EC barrier permeability. Sepsis is usually marked by increased vascular permeability resulting from a disrupted EC barrier due to changes in interendothelial junctions leading to leakage of water and proteins, resulting in tissue and organ damage (80). S1P promotes EC migration, reduces vascular permeability in sepsis, and plays an important role in maintaining EC barrier integrity (75, 81). The earlier studies have shown that S1P promotes cell migration during vascular development in zebrafish (82) and recent studies have demonstrated that S1P-S1PR1 signaling promotes cellular AJ by altering actin rearrangements in ECs, which results in the expression of molecules such as vascular endothelial cadherin (16). Liu et al. (83) hypothesized that activation of S1PR1 by S1P has a proangiogenic effect through experiments in mice in which S1PR1 knockout mice developed embryonic hemorrhage leading to intrauterine death, which was supported by studies demonstrating reduced vascular permeability after administration of exogenous S1P (84). The severity of the impairment of endothelial function in sepsis increases with a decrease in S1P concentration in the plasma (85). S1P participates in the sepsis disease process by maintaining the vascular endothelial barrier (16, 75).

In addition, S1P has been shown to have contractile effects in arteries of a variety of animals (86), angiotensin II (AngII) treatment leads to hypertension (BP) associated with increased plasma S1P and circulating T cell counts, and SphK2 activity is critical for AngII-induced lymphocyte trafficking. Moreover, the dysregulation of SphK2 expression is related to the thrombotic inflammatory phenotype of microvessels and the functional changes of small resistance arteries, leading to the development of hypertension (87). Ang II infusion can cause S1P to convert PRR into soluble PRR (sPRR) in the cells, which eventually promotes the increase of blood pressure through various reactions. However, the S1P inhibitor PF429242 could not achieve the pressor effect by blocking the release of sPRR and the transformation of Ang I. The above results indicate that S1P and its signaling axis play an important role in blood pressure regulation (88, 89).

### 4.2. S1P promotes multiple immune cell trafficking in sepsis

Sepsis process is divided into two immune phases, including an initial immune activation phase, followed by an immunosuppressive phase that culminates in immune cell death (90). Cells of both the innate and adaptive immune systems play key roles in the host response to sepsis (91). In the early stage of sepsis, necrotic tissues and microorganisms release damage-associated molecular patterns (DAMPs) and pathogen-associated molecular patterns (PAMPs), leading to rapid activation of pattern recognition receptors (PRRs), including Toll-like receptors expressed by cells of the innate immune system (92), macrophages, dendritic cells. Neutrophils are stimulated to undergo hyper proliferation and clear the outbreak of infection from the body as soon as possible. Innate immune activation is followed by activation of the adaptive immune system, leading to T cell receptor activation of T helper and cytotoxic T cells, with cell differentiation and proliferation, resulting in a specific adaptive immune response. In addition, immune cells produce and release large amounts of inflammatory mediators, such as IL-1β, IL-2, IL-6, TNF-α, and chemokines, as well as cytokines acting on the ECs to increase vascular permeability. During sepsis, local inflammation gives way to a systemic response, leading to a widespread infection resulting in an inflammatory syndrome (91). Extensive vasodilation leads to hypoperfusion and tissue hypoxia, causing disseminated intravascular coagulation and MODS (93).

Sphingosine 1-phosphate is considered a circulating marker that signals immune cells to help them find blood and lymphatic vessels, and signals the ECs to stabilize the vasculature (94). S1P is involved in T cell migration and promotes the development, differentiation, trafficking, and other processes of B cells, NK cells, and neutrophils. S1P is distributed at various sites in varying concentrations, and in immune responses S1P follows a concentration gradient, transporting T cells from lymph node egress (low concentration) into lymph fluid (high concentration), and ultimately through the blood into the infected tissues, thereby defending against foreign pathogen invasion (24). As early as 2004, it has been demonstrated that S1PR1-deficient mice lack T cells in the peripheral blood and are unable to deliver mature T cells to the blood (95). S1P-S1PR1 signaling was subsequently found to regulate T cell migration and was found to be involved in the regulation of B cells, NK cells, and also the development and differentiation of leukocytes, antigen-presenting cells, and other immune responses (96). When lymphocytes are stimulated by inflammation, which temporarily shuts down the lymphoid organ egress of lymphocytes, the activation marker CD69 binds S1PR1 and promotes S1P internalization to exert anti-inflammatory effects. Also, the presence of an S1P concentration gradient *in vivo* enables the T cells to stay in the lymph nodes for an extended period of time, and allows them to remain at the inflammatory site, and does not affect transcription, translation, and modification (24, 97). Most immune cells express S1PR1, S1PR2 activates Rho and inhibits migration, confines cells to germinal centers, and regulates B cell migration and positioning (98), S1PR5 regulates NK cell egress and NK cell localization in LNs, favoring bacterial clearance (99), whereas the other receptors are restricted to the expression pattern of immune cell subsets. Moreover, a recent study has demonstrated that S1PR5 inhibits the formation of Tissue-resident memory T (TRM) cells and blocking S1P signaling in TRM is beneficial for the treatment of chronic inflammation (100). S1P induces chemotaxis of immature cells and regulates cytokine release in mature human dendritic cells (DCs) to generate Th2 immune responses (101).

### 4.3. S1P pathway suppresses sepsis-induced inflammatory factor storm

SphKs-S1P-S1PRs signaling suppresses sepsis-induced cytokine storm and provides a new potential therapeutic target for sepsis treatment (102, 103). Inflammatory factor storm is a major cause of mortality in patients suffering from severe sepsis. The mechanism involves an over activation of immune cells, causing release of a large amount of inflammatory factor in the cells, which strongly attack the infected cells to cause MODS. Prevention of the inflammatory factor storm has always been a critical aspect in the field of sepsis research (104). S1PR1 agonist CYM5442 markedly suppressed the exaggerated inflammatory response upon H1N1 influenza challenge even in the early H1N1 influenza phase, providing *in vivo* protection with reduced mortality (103). Several studies have demonstrated reversal of COVID-19 complications and improved survival by modulation of SphKs and S1P-S1PR pathways (103, 105) in the recent outbreak of COVID-19 pandemic, also characterized by an inflammatory storm, hyperinflammation and septic shock (106, 107). A recent report proposed that the lower the S1P level in the blood, the more severe was the condition of the COVID-19 patient (108). S1P exerts opposite effects by binding to different receptor, and when S1P binds to S1PR1, it inhibits TNF-α responses, to produce anti-inflammatory effects (109).

## 5. S1p signal pathways and barrier homeostasis in the gut

### 5.1. The intestinal barrier in intestinal injury in sepsis

The intestinal barrier function consists of three main lines of defense. Firstly, the biological barrier, which consists of the normal intestinal flora (gut microbiota). The microbiota not only inhibits the growth of potentially pathogenic bacteria, but also exerts important metabolic, immune, and gut protective functions such as synthesizing energy substances required by the intestinal epithelium to produce PAMP for promoting gut immune regulation and interacting with the immune system (110). Secondly, a mechanical barrier, consisting of IECs and capillary ECs, with an intact intestinal mucosal epithelium and TJs between cells, which restricts the passage of ions, molecules, and cells through the paracellular space (9). The epithelial cells are not only involved in the direct defense against microorganisms, but also send signals to the mucosal immune system by producing cytokines and chemokines (111). Thirdly, the chemical barrier consisting of gastric acid, bile, trypsin, lysozyme, and intestinal fluid in the GI tract, as well as several organic acids produced by the gut flora (112). Fourthly, the immune barrier, composed of gut associated lymphoid tissue (GALT), T cells, B cells, innate lymphoid cells, and macrophages and dendritic cells in the lamina propria, which interacts with the microbiota to combat the invasion of foreign substances from the gut (113).

The epithelium, immune system, and microbiome are essential for maintaining homeostasis in the gut (114). Any irregularities in these can cause dysregulation of the immune system, interruption of intestinal epithelial homeostasis, uncontrolled bacterial colonization, and epithelial barrier dysfunction, leading to intestinal damage (10, 115). Under the stress state of severe infection and inflammatory response, the protective function of the intestinal mucosal barrier is weakened, and then the intestinal bacterial translocation occurs. Endotoxin, bacteria and antibody mediators continue to enter the blood and lymph, leading to the release of a variety of inflammatory mediators, inducing the production of a variety of cytokines, activating the inflammatory cascade and activating the acquired immune system. As a result, the body cannot effectively regulate the regulation of inflammation and immune response, which has become the key factor of MODS in sepsis patients (116, 117). Septic shock is characterized by endothelial glycocalyx breakdown and endothelial damage, leading to fluid extravasation, organ failure, and death. Several studies have pointed out that endothelial damage plays a critical role in sepsis-induced organ failure (118). Therefore, alteration of intercellular junction proteins leading to the disruption of intestinal membrane permeability is a critical factor in sepsis pathogenesis and can serve as an early marker in the development of MOF.

### 5.2. S1P maintains the intestinal barrier in septic intestinal injuries

Intestinal function mainly includes food digestion and absorption, material exchange and metabolism, preventing bacterial translocation, and maintaining an intact intestinal mucosal barrier, which is important with regards to sepsis (119). SM regulates the proliferation and migration of intestinal mucosal epithelium by participating in immune activation and participating in pro- and anti-inflammatory responses (15). The sphingolipids in food are digested and absorbed in the intestine to generate Sph and S1P, SLs and their metabolites alleviate inflammation by altering the intestinal microenvironment, protecting the intestinal barrier, and activating anti-inflammatory pathways (120–122).

**FIGURE 3:**
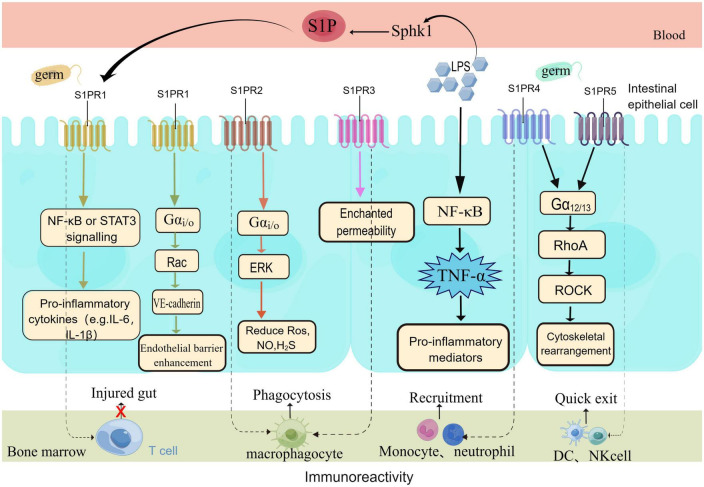
S1P protects the intestinal barrier through multiple means. S1P protects the intestinal barrier in multiple ways. Under the stimulation of pathogens, the body rapidly activates the inflammatory pathway and produces a large number of inflammatory factors. The body’s immune response is unbalanced in sepsis, according to the different signaling pathways of S1PRs, barrier protection is exerted to restore body homeostasis. Endothelial cells mainly express the S1PR1-3 receptor signal, with the highest expression of S1PR1, which plays a major role in barrier protection. At the same time, the five receptors are involved in the migration of a variety of immune cells (T cells, B cells, macrophages, neutrophils, DC, NK cells.

Inflammatory stress in sepsis can cause intestinal epithelial apoptosis, intestinal injury and translocation of bacterial toxins, which can lead to intestinal organ dysfunction (123). Studies have found S1P to be a barrier enhancing molecule, in sepsis intestinal injury. S1P mainly operates through one of the following three mechanisms (Figure 3, By Figdraw): (1) promotes intercellular junctions strengthens the barrier of the intestinal ECs; (2) involved in the survival, differentiation, migration, and proliferation of a variety of immune cells in the gut; (3) reduces damage caused by the gut microbiome, maintains the barrier function of the intestinal epithelium thus alleviating sepsis-associated intestinal injury, thereby improving sepsis survival (16, 19, 118, 124). The mechanism of intestinal injury is mainly hemodynamic. Cellular alterations, and bacterial toxins cause intestinal edema and intestinal dysfunction by disrupting normal intercellular junctions, reducing vascular endothelial cadherin and TJ and increasing vascular permeability (13). S1P acts on the receptors S1PR1-3 present on the ECs to activate downstream signaling pathways, reduces the production of reactive oxygen species (ROS), nitric oxide (NO), and hydrogen sulfide (H2S), and reduces vascular permeability to maintain the EC barrier (125, 126). It is important to note that at the level of AJ, S1PR1 is an important vascular barrier protective mechanism (127). In addition, the binding of S1P-S1PRs has been reported to induce NO production and inhibit ROS production thereby exerting vascular protection effects with the participation of HDL (128). Vascular leakage is a feature of severe sepsis and SIRS, that can lead to a distributive S1P can preserve endothelial function, induce tight junction formation, and prevent vascular leakage (129). In a clinical trial, it was found that the plasma concentrations of syndecan-1 (SYN-1) and VE-cadherin (endothelial cell junctions) in septic shock patients significantly increased within 7 days, patients with more severe diseases have higher concentrations of SYN-1 and VE-cadherin in their plasma, while lower levels of S1P, demonstrate a close correlation between S1P and endothelial damage in patients with septic shock (118). In addition, studies have shown that the lower the S1P level, the higher the SOFA score, and the more severe the condition, S1P levels can predict the mortality rate of sepsis (56). Transgenic mice with low plasma S1P levels are more susceptible to lipopolysaccharide (LPS) infection as compared to wild-type mice. FTY720 or S1P treatment can inhibit vascular leakage and inflammatory responses in mice or rabbits subjected to LPS treatment (75).

Sphingosine 1-phosphate concentrations in tissues increase in the early stage of the inflammatory response and inhibit the entry of T cells into lymphocytes in intestinal tissues, S1P levels in tissues reduce during the resolution of inflammation (130). S1P-S1PRs signaling has multiple effects, and S1PR1 inhibits the trafficking of T lymphocytes to inflamed tissues (96), thus alleviating intestinal injury. S1PR1 is also involved in and promotes macrophage recruitment, apoptosis and anti-inflammatory responses and dendritic cell trafficking (131). S1PR2 enhances the phagocytosis of macrophages and regulates mononuclear cell migration by reducing IFN- γ induced intestinal mucosal permeability damage (132). However, S1PR3 causes increased mortality in sepsis patients by delaying the maturation of phagosomes in macrophages (133). S1PR4 regulates monocyte and neutrophil recruitment, and S1PR5 is expressed by dendritic cells and natural killer cells, accelerating the withdrawal of natural killer cells from bone marrow and monocyte transport (134). Research has found that the alkaloid berberine, found in plants is associated with the metabolism of S1P and the regulation of intestinal immune response and inflammation related pathology; S1P regulates intracellular calcium levels, cell movement, proliferation, and apoptosis to maintain gastrointestinal immune homeostasis (15). In addition, nuclear peroxisome proliferator-activated-receptor-γ (PPAR-γ) is widely expressed in immune cells and ECs, and its main function is to sense intracellular nutrient concentration and regulate the expression of the gene involved in maintaining metabolism and immune homeostasis (135). PPAR-γ can weaken the inflammatory response by enhancing the anti-inflammatory effect of dietary SM (120). PPAR- γ is expressed by CD4^+^T cells, but a selective S1P1 receptor modulator significantly reduces CD4^+^T cells on lymphocyte subsets in healthy humans (136). Studies have indicated that increased expression of PPAR-γ reduces ROS levels and inhibits the TXNIP/NLRP3 signaling pathway, thereby reducing pyroptosis and liver dysfunction during sepsis (137).

Sepsis seriously affects the composition of the intestinal microbiota, leading to organ failure (138). The intestinal microbiome plays an important role in intestinal injury. Bacterial toxins are released from the infection site into the intestinal tract. Bacterial translocation alters the intestinal microenvironment, and the imbalance in intestinal microbiome flora aggravates intestinal injury (8, 138). Previous research has demonstrated that the activation of SphKs-S1P-S1PR signaling in intestinal inflammation can be correlated to the imbalance in intestinal microbiota, and the corresponding signaling pathway can defend against bacterial toxins and protect the intestinal mucosal barrier (139). At the same time, the gut microbiota is crucial for the development and maturation of the immune system, and the host’s immune response can be induced by the imbalance of the gut microbiota. Detection of PAMPs innate immune cells through pattern recognition receptors constitutes the first stage of host response. The endotoxin (LPS), and berberine, which is a commonly used drug for treating intestinal infections, can enhance the expression of S1P and subsequently the downstream signaling pathways, reducing LPS-induced intestinal injury (140).

### 5.3. S1P signaling pathway in the intestine

The gut plays a central role in the progression of sepsis (8). Sphingolipid signaling, particularly the signal transduction between tumor necrosis factor alpha (TNF-α) and NF-κB involved in S1P is considered as one of the mediators promoting gastrointestinal inflammation (141, 142). S1PR, which is expressed in all gastrointestinal tissues, activates classic inflammation related transcription factors. TNF-α is a major pro-inflammatory cytokine that activates the downstream target SphKs-S1P axis, which is the most commonly studied mechanism of intestinal inflammatory diseases. The TNF- α downstream pathway is the target for the treatment of inflammation related intestinal injury (143, 144). TNF-α signaling is a characteristic to proinflammatory cytokines in innate immunity, which induces the expression of inflammatory genes, and directly drives the inflammatory response to induce cell death (145). Research indicates that S1P affects the generation of lymphatic vessels by promoting the secretion of TNF-α and IL-1β through the NF-κB signaling pathway mediated by S1PR1 (146). Berberine can enhance S1P expression by regulating ApoM-S1P and inhibiting the NF-κB pathway, thereby protecting the damaged intestinal vascular barrier (140). The reason why S1PR-targeted modulators can specifically treat inflammatory bowel disease which is one of the several autoimmune diseases is mainly associated with the TNF-α pathway (147). In addition, the experimental results indicate that the MHC-II expression is inhibited by the S1P-S1PR2 axis in IECs through extracellular signal-regulated kinase activity, promoting CD4^+^T cell proliferation and protecting intestinal barrier function (148). The S1PR agonist FTY720 reduces vascular leakage and thus maintains the integrity of the barrier, and exerts a protective effect on the inflammatory cascade response caused by mesenteric stress (149). At the same time, a direct relationship was found to exist between gut Th17 cells induced by microbiota and peripheral injury, and the mechanism is that TNF T cells and Th17 cells rely on S1PR1 for excretion from intestinal lymphatic tissue (150).

## 6. S1P can be an intervention target for gastrointestinal injury in sepsis

A growing number of studies have demonstrated that S1P plays an important role in the vasculature, inflammatory response, immune response, and maintenance of the intestinal mucosal barrier, and is considered a potential therapeutic biomarker for sepsis. This might provide a new direction for the development of targeted therapeutics for sepsis by targeting the S1P-S1PR signaling mechanism (Table 1) (19, 126). Four S1PR modulators (fingolimod, siponimod, ozanimod, and ponesimod) have regulatory approval for multiple sclerosis. Targeting S1PRs for the treatment of inflammatory diseases has shown successful results in clinical trials, and they are mainly indicated for treating inflammatory bowel diseases (IBD), including ulcerative colitis and Crohn’s disease (151). The immunomodulator FTY720 is a structural analogue of S1P and acts in its phosphorylated isoform as an unselective agonist on S1PR1 and S1PR3-5 and a selective functional antagonist on S1PR1 (152). Fingolimod (FTY720), the most investigated modulator, is efficacious on all subtypes except S1PR2. It inhibits S1PR1 expression after activation of S1PR1 on lymphocytes and limits the egress of lymphocytes from lymphoid tissues, thus reducing lymphocytic infiltration and reducing the extent of damage, and ameliorating local inflammation in the intestine in murine colitis (17, 18). FTY720 improved sepsis incidence and reduced sepsis-induced hypothermia and weight loss. Lymphopenia leads to the accumulation of lymphocytes in peripheral lymph nodes and reduces the bacterial load in the liver, and proinflammatory factors such as plasma IL-6, TNF-α, MCP-1, and IL-10 are significantly reduced (153). In acute lung injury, sepsis-associated encephalopathy and cardiac function injury, FTY720 can be seen to show a protective phenotype in experimental sepsis by regulating vascular and immune functions (154–156). In the latest DSS-induced colitis model study, it was confirmed that FTY720 improved abnormal immune response by capturing T cells and inhibiting the polarization of M1 macrophages in mice with colitis, and this effect has not been clarified in sepsis (157). Previous studies reported that S1P regulates peritoneal B cell trafficking and subsequent intestinal IgA production, and FTY720 significantly reduced the production of natural gut secretory IgA derived from peritoneal B cells, demonstrating the critical role of S1P in intestinal cellular immunity (158). This study found that there is the production of Th17 proinflammatory cells during intestinal inflammation, and FTY720 directly inhibited the differentiation of Th17 cells *in vitro*, while increasing Treg differentiation from naive CD4 + T cells and inhibiting IL-23-mediated activation of STAT4, NF-kB and AKT. In addition, it also inhibited Dectin-1 expression in mature and immature monocyte-derived dendritic cells, which in turn inhibited curdlan-mediated production of IL-23p19, p40, IL-6, and IL-1β cytokines (159). Lymphopenia (T and B cells) induced by FTY720 did not negatively affect mortality from sepsis during challenge with severe abdominal sepsis (160).

**TABLE 1:** S1P modulators under development or in clinical testing.

Drug name	Target	Mechanisms	Disease	Influence	Current trial phase	Status
Fingolimod (FTY720)	S1PR1, 3, 4, 5	Functional “antagonist” and “agonist”	RRMS	Restrict lymphocyte egress from the lymph nodes; Reduce inflammatory cell infiltration of the central nervous system.	Marketed	
			Transplant	Decrease circulating alloantigen-reactive T cells without permanently destroying these cells	Phase 3	Completed
			Stroke	Agonist: Mitigate microvascular dysfunction; Agonist: Protect Blood-Brain-Barrier Functions; Agonist: Inhibit of autophagic pathways Agonist: Anti-inflammatory; Antagonist: Lymphopenia	Phase 2	Completed
			CIPD	Reduction in circulating memory T and B cells and increase in regulatory B cells.	Phase 3	Completed
			PPMS	Receptor internalization and degradation; Inhibit lymphocyte migration from lymph nodes	Phase 3	Terminated
			ALS	Decrease circulating lymphocytes	Phase 2	Completed
			Asthma	Reduce antigen-induced airway inflammation; Hyperresponsiveness	Phase 2	Completed
			Sepsis	Maintain the vascular endothelial barrier; Reduce secretion of pro-inflammatory cytokine	NO	
Siponimod	S1PR1, 5	Functional “S1PR1 antagonist” and “S1PR5 agonist”	SPMS	Reduce lymphocyte egress from lymph nodes; Inhibit lymphocyte migration from the periphery to the central nervous system	FDA approved	
			RRMS		Phase 2	Completed
			Active dermatomyositis	Participate in immunity	Phase 2	Terminated
			Hemorrhagic stroke	Anti-inflammatory and Neuroprotective	Phase 2	Completed
			Hepatic impairment	Siponimod were well tolerated with different levels of hepatic impairment	Phase 1	Completed
Ozanimod	S1PR1, 5	Functional “antagonist”	RMS	Induce lymphopenia by preventing lymphocyte egress from lymph nodes	FDA approved	
			UC	Induce peripheral lymphocyte sequestration and reduce circulating lymphocyte counts in the gastrointestinal tract Antagonist: Reduce clinical and histopathological severity of experimental colitis in animal models of human ulcerative colitis and Crohn’s disease	Phase 3	Recruiting
			Crohn		Phase 3	Recruiting
			COVID-19	Inhibit the inflammatory pathway	Phase 2	Terminated
Ponesimod	S1PR1	Functional “S1PR1 agonist”	MS	Internalization of the receptors and inhibit the egress of lymphocytes from lymph nodes; Reduce the number of lymphocytes in peripheral blood; Prevent lymphocytes circulation to sites of inflammation	FDA approved	
			Psoriasis		Phase 2	Completed
ABC294640	SphK2	Functional “SphK2 antagonist”	Cancer and Solid Tumors	Inhibit the growth of tumor cells by blocking some of the enzymes needed for cell growth	Phase 2	Completed
Safingol	SphK1	Functional “SphK1 antagonist”	Locally advanced or metastatic solid tumors	Inhibit the growth of tumor cells by blocking the enzymes necessary for cell growth and by stopping blood flow to the tumor.	Phase 1	Completed
Cerenimod	S1PR1	Functional “S1PR1 agonist”	SLE	Internalization of the receptors and induce a long-lasting inhibition of the egress of lymphocytes from lymphoid organs	Phase 2	Completed

RRMS, relapsing remitting multiple sclerosis; CIPD, chronic inflammatory demyelinating polyradiculoneuropathy; PPMS, primary progressive multiple sclerosis; ALS, amyotrophic lateral sclerosis; SPMS, secondary progressive multiple sclerosis; RMS, relapsing multiple sclerosis; UC, ulcerative colitis; SLE, systemic lupus erythematosus.

The discovery of the S1PR3 receptor mediating bradycardia in mice prompted the search for modulators devoid of S1PR3 signaling (161). The published study demonstrating that the Sphingosine 1-phosphate receptor modulator, siponimod, does not just ameliorate the inflammatory aspect but also the degenerative aspect of secondary progressive MS (162).

Ozanimod, which attenuates immune responses by reducing circulating lymphocytes, has been shown to reduce inflammation and disease severity in animal models of colitis (163). Phase III trials are currently being conducted to test its clinical efficacy in ulcerative colitis, clinical trials on Ozanimod intervention in COVID-19 are also ongoing (25, 164, 165), Furthermore, under conditions of non-interference with protective immunity, targeting specific T lymphocyte subsets of autoimmune disease by ozanimod improves the disease situation (166). Recently, ponesimod another receptor modulator has been highlighted for its safety and efficacy by the continuous research on autoimmune diseases, which specifically activate signal transduction through the S1PR1 pathway (167). Ponesimod can prevent inflammation due to autoimmune reactions and can also cross the blood-brain barrier hindering synaptic neurodegeneration, thus exerting neuroprotective behavior (168, 169).

Since S1P exhibits multiple protective effects in septic intestinal injury, the fatality rate associated with septic intestinal injury can be reduced by elevating S1P levels or using S1PRs modulators (19). With regards to the experimental observation of lowered S1P levels in the plasma of patients with sepsis, some studies have demonstrated that intravenous injection of exogenous S1P into mice caused a transient increase in plasma S1P levels. But this exogenous S1P was cleared out within 15–30 min, because of the short biological half-life of S1P. It was also observed that the S1P degradation was related to the cellular location since intracellular S1P was degraded by SPL, while the extracellular degradation was not related to SPL (170). Treatment of sepsis by direct injection of S1P is not yet feasible, and increasing S1P levels in plasma may either enhance S1P generation by SphKs, or reduce S1P degradation (19). It is important to explore the mechanism through which S1P functions through the immune response for the treatment of sepsis (24). Based on current clinical data on S1PRs modulators, they are thought to reduce inflammation in immune-mediated diseases mainly by blocking lymphocyte egress from lymph nodes to the bloodstream (151, 171). To date, S1PR modulators have made great progress in the treatment of autoimmune intestinal inflammatory diseases, and some molecules have already been approved for use with proven safety and efficacy (169), however, the efficacy of these agents in treating sepsis-associated intestinal injury requires further investigation (23). In addition, the present study found inter-individual functional differences between SphKs and S1PRs, for example, SphK2 inhibits the production of anti-inflammatory factor IFN-β, in contrast to action exerted by SphK1 (102) and S1PR1 exerts anti-inflammatory effects, whereas S1PR2 accelerates the induction of TNF-α activation, which promotes inflammatory responses (172, 173). Therefore, there is a continuous effort to develop new drugs that target the function of different kinases with receptor subtypes, with the aim of providing new therapeutic targets for sepsis-associated intestinal injury (23).

## 7. Conclusion and perspectives

Sphingosine 1-phosphate as one of the EC barrier reinforcing molecules plays an important role in maintaining barrier integrity. In the pathogenic process of sepsis, S1P reduces the case fatality rate associated with sepsis by suppressing the storm of the inflammatory factor. The gut, as the prime factor in MODS, is often attacked by sepsis bacteria with toxins, causing sepsis-associated intestinal injury and dysfunction. S1P binds to different S1PRs to play different roles, for example, the S1P-S1PR1 pathway regulates multiple immune responses and decreases inflammatory responses and is an effective target for the treatment of sepsis. In intestinal injury, S1PR1 alleviates the inflammatory storm and is involved in diverse immune cell migration. The complex regulatory network of S1P signaling, metabolism, and trafficking have made it difficult to translate the present findings translated into clinical applications. Understanding the mechanisms by which intestinal inflammation, immune responses, and S1P interact could help in the development of novel targets with an aim to reduce the morbidity and mortality associated with intestinal injury in sepsis. In the future we need to explore the signaling pathways of S1P on individual S1PRs in the gut under sepsis conditions and observe the mechanism by which the gut microbiota regulates S1P levels. Whether S1PR modulators can be effective targets for intestinal injury in sepsis requires further study. Because of the short biological half-life of S1P, the current available experimental evidence does not support the direct injection of S1P. The currently developed treatment modalities involving receptor modulators show good stability versus effectiveness. Developing highly selective modulators of S1PRs are the future research directions. Finally, the mechanisms of S1P signaling could provide a high-value regimen superior to traditional therapy for combating sepsis-associated intestinal injury, providing a new avenue for exploring the pathogenesis of the disease and implementing individualized therapeutic strategies.

## Author contributions

GS: Writing—original draft. BW: Writing—original draft. HZ: Writing—review and editing. JY: Writing—review and editing. XL: Writing—review and editing.
